# Evaluation of the Rapid Antigen Detection Test for Diagnosing SARS-CoV-2 during the COVID-19 Pandemic: Experience from a Centralized Isolation Site in Shanghai, China

**DOI:** 10.1128/spectrum.04542-22

**Published:** 2023-01-19

**Authors:** Xu Zhong, Li Zhang, Di Ma, Yingying Shi, Jiayin Wu, Xiaosheng Xu, Weihong Chen, Beiwen Wu, Yiding Tang, Hongping Qu, Qing Xie, Yi Yang, Jialin Liu, Haiguang Xin

**Affiliations:** a Department of Infectious Diseases, Ruijin Hospital, Shanghai Jiao Tong University School of Medicine, Shanghai, China; b Department of Critical Care Medicine, Ruijin Hospital, Shanghai Jiao Tong University School of Medicine, Shanghai, China; c Department of Cardiovascular Surgery, Ruijin Hospital, Shanghai Jiao Tong University School of Medicine, Shanghai, China; d Department of Endocrine and Metabolic Diseases, Shanghai Institute of Endocrine and Metabolic Diseases, Ruijin Hospital, Shanghai Jiao Tong University School of Medicine, Shanghai, China; e Shanghai National Clinical Research Center for Metabolic Disease, Ruijin Hospital, Shanghai Jiao Tong University School of Medicine, Shanghai, China; f Department of General Surgery, Ruijin Hospital, Shanghai Jiao Tong University School of Medicine, Shanghai, China; g Operating room, Ruijin Hospital, Shanghai Jiao Tong University School of Medicine, Shanghai, China; h Department of Statistics and Information, Ruijin Hospital, Shanghai Jiao Tong University School of Medicine, Shanghai, China; i Department of Obstetrics and Gynecology, Ruijin Hospital, Shanghai Jiao Tong University School of Medicine, Shanghai, China; j Department of Nephrology, Ruijin Hospital, Shanghai Jiao Tong University School of Medicine, Shanghai, China; k Department of Nursing, Ruijin Hospital, Shanghai Jiao Tong University School of Medicine, Shanghai, China; University of Mississippi Medical Center

**Keywords:** SARS-CoV-2, rapid antigen detection test, diagnostic performance, nasopharyngeal swabs, upper respiratory tract symptoms

## Abstract

Rapid and reliable diagnosis is important for the management of individuals infected with severe acute respiratory syndrome coronavirus 2 (SARS-CoV-2). The rapid antigen detection test (RADT) is a rapid, inexpensive, and easy method. Several studies have reported that RADTs performed well in many countries; however, very few studies have been reported in China. In this study, we assessed the performance of the RADT (Ediagnosis COVID-19 antigen test kit). This study was conducted in a centralized isolation site in Shanghai and enrolled 716 patients with COVID-19 and 203 noninfected participants. Nasopharyngeal swabs from all participants were collected on the same day and tested using the RADT and real-time reverse transcription-PCR (RT-PCR). The performance of the RADT was evaluated in different scenarios, such as threshold cycle (*C_T_*) values, symptomatic phase, and symptoms on the day of testing. The results demonstrated that the sensitivity for patients with *C_T_* values lower than 20 was 96.55% (95% confidence interval [CI], 87.05 to 99.4). The sensitivities were 78.4% (95% CI, 69.96 to 85.05) for participants within 5 days after the first RT-PCR-positive result and 90.77% (95% CI, 80.34 to 96.19) within 5 days after symptom onset. Moreover, the sensitivity of the RADT was more than 80% for patients with symptoms on the day of testing, including fever (89.29%), cough (86.84%), stuffy nose (92.59%), runny nose (92%), sore throat (81.25%), and muscle pain (80.77%), especially for those with upper respiratory tract symptoms. The specificity of the RADT was good in all scenarios. During the SARS-CoV-2 epidemic, Ediagnosis performed excellently in individuals with a higher viral load (evidenced by lower *C_T_* values), individuals in the early symptomatic phase, and especially those with upper respiratory tract symptoms.

**IMPORTANCE** RADTs have demonstrated excellent performance in many counties for screening SARS-CoV-2 infection, but very few studies have been conducted in China. The performance of RADTs is largely related to different real-life scenarios. In our study, the performance of the RADT was evaluated in different scenarios, such as *C_T_* values, symptomatic phase, and symptoms on the day of testing. The results demonstrated that Ediagnosis (an RADT made in China) performed excellently for individuals with a higher viral load (evidenced by lower *C_T_* values), individuals in the early symptomatic phase, and especially those with upper respiratory tract symptoms.

## INTRODUCTION

The severe acute respiratory syndrome coronavirus 2 (SARS-CoV-2) variant Omicron was first detected in China on 11 November 2021 and then spread across Shanghai in the following months (1, 2). Previous studies have indicated that Omicron, a variant of concern (VOC), is more transmissible than the Delta variant (3, 4). Meanwhile, infection with Omicron often causes milder symptoms, especially in vaccinated individuals, which greatly hinders the early identification of infected patients (5, 6). The real-time reverse transcription-PCR (RT-PCR) assay, the current gold standard for laboratory diagnosis owing to its high sensitivity and specificity, requires at least 4 h of operation performed by skilled technicians and specialized equipment and reagents (7, 8). However, many countries have encountered shortages of RT-PCR reagents and specialized personnel. To control the spread of SARS-CoV-2 infection, an early and reliable diagnostic strategy is urgently required (9, 10). Currently, there are multiple commercial rapid antigen detection tests (RADTs) that can be performed within 20 min without requiring any specialized instruments and personnel, and their performance has been tested in several countries (11–14). This study aimed to determine the performance of Ediagnosis, an RADT approved for use in China in March 2022, in several real-life scenarios in Shanghai.

## RESULTS

### Patient population.

A total of 716 patients with COVID-19 were enrolled in this study. This represented 331 (46.23%) RT-PCR-positive and 385 (53.77%) RT-PCR-negative patients on the day of testing. A total of 61.31% (439 of 716) were male. The median age of the participants was 43 years (interquartile range [IQR], 31, 54). A total of 467 patients with COVID-19 completed a questionnaire about symptoms; of these patients, 420 (89.94%) had symptoms (Table 1). The most common manifestations were cough (62.96%), fever (39.61%), stuffy nose (35.33%), and sore throat (33.83%) (Table 1). Of the patients who completed the questionnaire, 19.27% had comorbidities, and the common comorbidities were hypertension (12.42%), diabetes (3.21%), and coronary heart disease (1.28%).

**TABLE 1:** Baseline characteristics of COVID-19 patients

Characteristic	Value for patients (*n* = 716)
Sex, male, no. (%)	439 (61.31)
Age, yrs, median (IQR)	43 (31, 54)
Filled out questionnaire, no.	467
No. (%) with symptoms	420 (89.94)
Fever	185 (39.61)
Cough	294 (62.96)
Stuffy nose	165 (35.33)
Runny nose	153 (32.76)
Sore throat	158 (33.83)
Itchy throat	136 (29.12)
Fatigue	126 (26.98)
Muscle pain	141 (30.19)
Diarrhea	68 (14.56)
Dysgeusia	38 (8.14)
No. (%) with comorbidity	90 (19.27)
Hypertension	58 (12.42)
Diabetes	15 (3.21)
Coronary heart disease	6 (1.28)

### Correlation of days after diagnosis and *C_T_* value.

**FIG 1:**
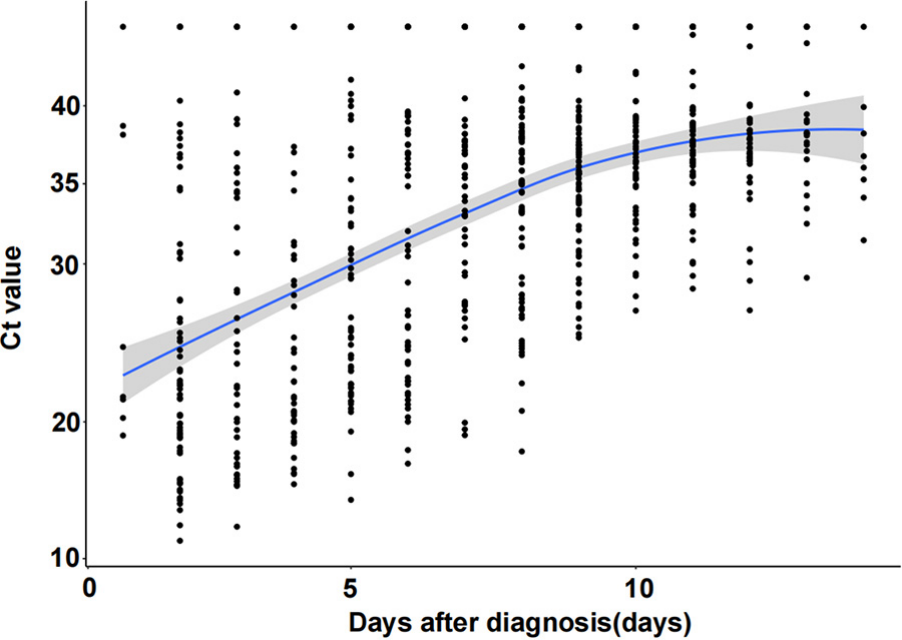
Relationship between days after diagnosis (days) and the *C_T_* value of RT-PCR assay (*C_T_* value of the SARS-CoV-2 open reading frame [ORF] or nucleocapsid protein [N], whichever was lower).

To capture the relationship between the days after diagnosis (days) and the threshold cycle (*C_T_*) value, a segmented linear regression model was used, including 669 patients with time postdiagnosis within 14 days (Fig. 1). The viral load showed a decreasing trend over time.

### Relationship between RADT performance and *C_T_* value in RT-PCR-positive patients.

**FIG 2:**
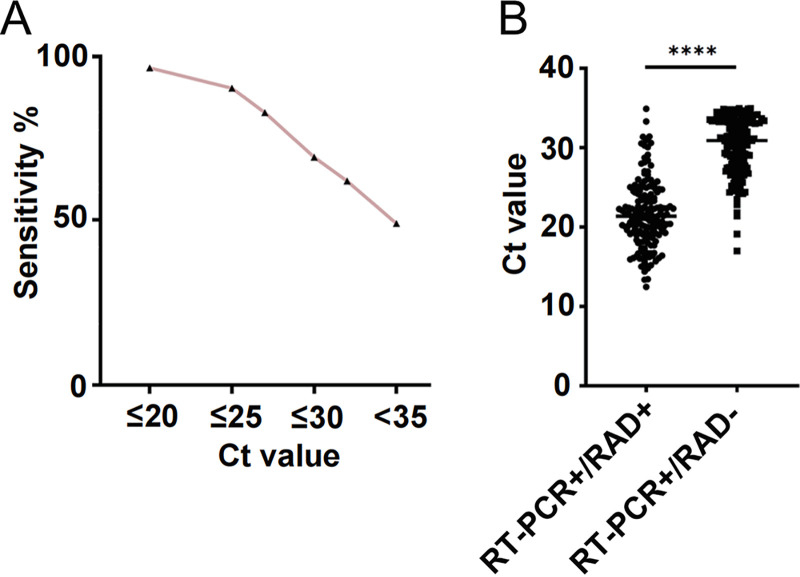
RT-PCR *C_T_* values and efficiency of RADT. (A) Sensitivity of the RADT according to the *C_T_* value; (B) distribution of the *C_T_* values for the RADT of nasopharyngeal swab specimens with positive and negative results (*C_T_* value of the SARS-CoV-2 open reading frame [ORF] or nucleocapsid protein [N], whichever was lower). ****, *P* < 0.0001.

As shown in Fig. 2A, the sensitivity of RADT increased in parallel with the SARS-CoV-2 RNA load, reaching 96.55% (95% confidence interval [CI], 87.05 to 99.4) in samples with *C_T_* values of ≤20. RADT sensitivity was 86.36% (95% CI, 77 to 92.45) with *C_T_* values of 20 to 25. Nevertheless, RADT sensitivity decreased to 8.26% (95% CI, 4.08 to 15.51) with *C_T_* values of 30 to 35. Among the pool of RT-PCR-positive samples, the median *C_T_* value of RADT-positive samples was lower than that of RADT-negative samples (21.39 versus 30.93; *P* < 0.0001) (Fig. 2B). RADT sensitivity was significantly reduced with a low viral load.

### Relationship between RADT sensitivity and symptoms and days after diagnosis.

To maximize sensitivity, the timing of RADT needs to be optimized for screening for SARS-CoV-2 in real-life settings. Of all 331 RT-PCR-positive patients, 163 (49.24%) were positive by the RADT and 168 (50.76%) were negative. The overall sensitivity was 49.24% (95% CI, 43.75 to 54.76). Based on the days after the first RT-PCR positivity and symptoms, further analysis was conducted to examine the RADT sensitivity. In patients within 5 days after diagnosis, the sensitivity was 78.4% (95% CI, 69.96 to 85.05). However, the sensitivity decreased to 51.02% (95% CI, 40.78 to 61.18) for patients 5 to 7 days after the diagnosis. The sensitivity was 61.64% (95% CI, 49.49 to 72.56) for patients 5 to 7 days after symptom onset and increased to 90.77% (95% CI, 80.34 to 96.19) within 5 days after symptom onset (Table 2). A total of 63 patients had symptoms on the day of testing with higher sensitivity (80.77% [95% CI, 67.03 to 89.92]), including 42 patients who tested positive by both RT-PCR and RADT. On the day of testing, the sensitivities of RADT for patients with fever, cough, stuffy nose, runny nose, sore throat, and muscle pain were 89.29%, 86.84%, 92.59%, 92%, 81.25%, and 80.77%, respectively (Table 2).

**TABLE 2:** Comparison of detection sensitivities of the RADT^*a*^

Patient group	No. of patients with indicated RADT result		Sensitivity, % (95% CI)
	Positive	Negative	
All patients	163	168	49.24 (43.75, 54.76)
Patients with indicated period after diagnosis			
<5 days	98	27	78.4 (69.96, 85.05)
5–7 days	50	48	51.02 (40.78, 61.18)
Patients with indicated period after symptom onset			
<5 days	59	6	90.77 (80.34, 96.19)
5–7 days	45	28	61.64 (49.49, 72.56)
Patients with indicated symptom on the day of testing	42	10	80.77 (67.03, 89.92)
Fever	25	3	89.29 (70.63, 97.19)
Cough	33	5	86.84 (71.12, 95.05)
Stuffy nose	25	2	92.59 (74.25, 98.71)
Runny nose	23	2	92 (72.5, 98.6)
Sore throat	26	6	81.25 (62.96, 92.14)
Muscle pain	21	5	80.77 (60.02, 92.69)

a All patients were positive by RT-PCR.

### Relationship between RADT specificity and *C_T_* value and days after diagnosis.

Of the 385 RT-PCR-negative patients, 378 (98.18%) tested negative and 7 (1.82%) tested positive according to the RADT. Specificity was 98.05% (95% CI, 95.24, 99.28) in samples with *C_T_* values of ≤40 and 98.45% (95% CI, 93.95 to 99.73) in samples with *C_T_* values of >40 (Table 3). The specificity of the RADT was 96.83% for patients 5 to 7 days after diagnosis and 98.92% for patients 7 to 14 days after diagnosis and increased to 100% more than 14 days after diagnosis (Table 3). Furthermore, there were 203 noninfected participants based on RT-PCR, and the whole population had been vaccinated with at least two doses of SARS-CoV-2 vaccines. The median age was 33 years (IQR, 28.5, 42.5), and 111 (54.68%) patients were women. All of them tested negative by the RADT (specificity, 100%).

**TABLE 3:** Comparison of detection specificities of RADT^*a*^

Patient group	No. of patients with indicated RADT result		Sensitivity, % (95% CI)
	Positive	Negative	
All patients	7	378	98.18 (96.12, 99.2)
Patients with indicated *C_T_* value			
≤40	5	251	98.05 (95.24, 99.28)
>40	2	127	98.45 (93.95, 99.73)
>35	7	378	98.18 (96.12, 99.2)
Patients with indicated period after diagnosis			
5–7 days	2	61	96.83 (88.01, 99.45)
7–14 days	3	275	98.92 (96.62, 99.72)
>14 days	0	39	100 (88.83, 100)

a All patients were negative by RT-PCR.

## DISCUSSION

According to previous studies, the sensitivity of other RADTs varied between 24% and 93% in different populations and countries (15), but very few studies have been conducted in China. In this single-center cross-sectional retrospective study, after participants were diagnosed and admitted to the hospital, nasopharyngeal swab specimens were obtained from each participant by trained personnel on the same day and then used to perform the RADT and RT-PCR, to assess the performance of RADT.

The continual global circulation of SARS-CoV-2 is fueled by the appearance of the VOC Omicron, resulting in a pandemic in Shanghai (16). In many countries, RADTs for screening SARS-CoV-2 have contributed to early diagnosis. Thus, several commercially available RADTs have been used in Shanghai to reduce the screening time as much as possible. To date, very few studies conducted in China have reported the performance of RADTs. Our study aimed to evaluate the performance of the Ediagnosis COVID-19 antigen test kit in the detection of SARS-CoV-2 in different real-life scenarios. In this study, the sensitivity of RADT was 96.55% (95% CI, 87.05 to 99.4) in samples with *C_T_* values of ≤20. However, the diagnostic performance of the RADT was evaluated with an overall sensitivity of 49.24% (95% CI, 43.75 to 54.76), which resulted from the lower sensitivity (8.26%) in samples with *C_T_* values in the range of 30 to 35. Nordgren et al. reported relatively low sensitivities in samples with *C_T_* values over 30, 13% and 30.4% for the Panbio and Orient Gene tests, respectively (17). The sensitivity of the RADTs was directly related to the SARS-CoV-2 RNA load in the specimens (18, 19), and sensitivity increased when the viral load was high or the *C_T_* value was low (*C_T_* values ≤ 26.7, equivalent to ≥1E6 SARS-CoV-2 copies/mL [20]). Our results demonstrated that the viral load showed a decreasing trend over time, and the mean *C_T_* value was over 30 on day 6 after diagnosis (Fig. 1). Singanayagam et al. observed a strong relationship between *C_T_* value and virus isolation, and the estimated odds ratio (OR) of recovering infectious virus decreased by 0.67 for each unit increase in *C_T_* value (95% CI, 0.58 to 0.77) (21). Taken together with previous studies (13, 21), the results of our study indicate the impact of the *C_T_* value on RADT sensitivity with a decreasing trend in viral load over time.

Diao et al. reported that a proportion of nucleoprotein antigen detection assay-positive samples were from patients who had fever, fatigue, or cough onset within 1 day (22). Our results also showed that testing by an RADT at an early stage could improve sensitivity, such as early after symptom onset, which was consistent with previous studies (11, 13, 21). However, these studies did not investigate the relationship between RADT sensitivity and symptoms on the day of testing. In this study, the sensitivity was 80.77% for patients with symptoms on the day of the RADT. We found that the RADT had a higher sensitivity for patients with fever, cough, stuffy/runny nose, sore throat, myalgia, and fatigue on the day of testing. Fever is a common symptom of COVID-19 (23, 24). As previously described, nasopharyngeal swab viral loads for patients with fever tended to be significantly higher than those for patients without fever (25). Our results also concurred with these results. In our study, sensitivity was 89.29% for patients with fever. Preliminary data indicated that the Omicron variant predominantly replicated in the upper respiratory tract, resulting in upper respiratory tract symptoms, such as runny nose, sneezing, and sore throat (26). However, the relationship between RADT sensitivity and symptoms in the early stages of infection has not yet been determined. Our results showed that the sensitivity of the RADT was >80% for patients with upper respiratory tract symptoms, such as stuffy nose, runny nose, cough, or sore throat, on the day of testing. The results of our study also indicated that patients with upper respiratory tract symptoms on the day of testing may greatly benefit from the high diagnostic efficiency of the RADT.

The present study has several limitations. First, this was a single-center study with a limited sample size; thus, the confidence interval of sensitivity was relatively wide. Moreover, there were 249 patients with COVID-19 who did not participate in the questionnaire; therefore, their clinical characteristics related to the symptoms were not available, which affected the sample size to some extent.

In summary, this study showed that the performance of the Ediagnosis COVID-19 antigen test kit was consistent with that reported in many foreign research reports, and our results demonstrated that the RADT performed excellently for individuals with a higher viral load (evidenced by lower *C_T_* values), individuals in the early symptomatic phase, and especially those with upper respiratory tract symptoms.

## MATERIALS AND METHODS

### Study population and specimen collection.

This single-center cross-sectional retrospective study enrolled 716 patients with COVID-19 who were admitted to the North Branch of Ruijin Hospital (Shanghai, China) from 17 March to 22 April 2022. A total of 203 noninfected participants were enrolled as controls. The first RT-PCR-positive result was the time point at which the patient was diagnosed with COVID-19 using RT-PCR assay (*C_T_* value lower than 35) from 17 March to 22 April 2022. Each patient may have been diagnosed at a different time. After being diagnosed and admitted to the hospital, nasopharyngeal swab specimens were obtained from each participant by trained personnel on the same day and then used to perform the RADT and RT-PCR assay. The RADT was conducted immediately, and the RT-PCR assay was performed within 4 h following specimen collection. Baseline information of all participants was electronically extracted from the electronic health records (EHRs), including sex, age, and *C_T_* values from the RT-PCR assay. Clinical characteristics related to the symptoms and comorbidities (including hypertension, diabetes, and coronary heart disease) were collected using a questionnaire administered to patients with COVID-19. Written informed consent was obtained from all participants. This study was approved by the Ruijin Hospital Ethics Committee of Shanghai Jiao Tong University School of Medicine (approval number 2022-74) and was conducted in accordance with ethical principles that have their origin in the Declaration of Helsinki.

### RT-PCR assay for detection of SARS-CoV-2 RNA.

One nasopharyngeal swab specimen per participant was used for molecular diagnostics (RT-PCR) according to the standard procedures for COVID-19 diagnosis. In this study, the 2019-nCoV real-time PCR kit (Lifesaver, Shanghai, China) was used.

### Rapid antigen detection test.

After nasopharyngeal swab specimens were collected, RADT was performed immediately using the Ediagnosis COVID-19 (SARS-CoV-2) antigen test kit (Wuhan EasyDiagnosis Biomedicine Co., Ltd., Hubei, China) according to the manufacturer’s instructions. Briefly, the nasopharyngeal swab specimen was immersed 10 times in the sample preparation mixture provided in the kit. Then, 120 μL (3 drops) of the mixture was dropped into the well of the test device. After 15 min of incubation, the result was read in the device window: one line (C) indicated a RADT-negative result, and two lines (C and T) indicated a positive result.

### Statistical analysis.

**FIG 3:**
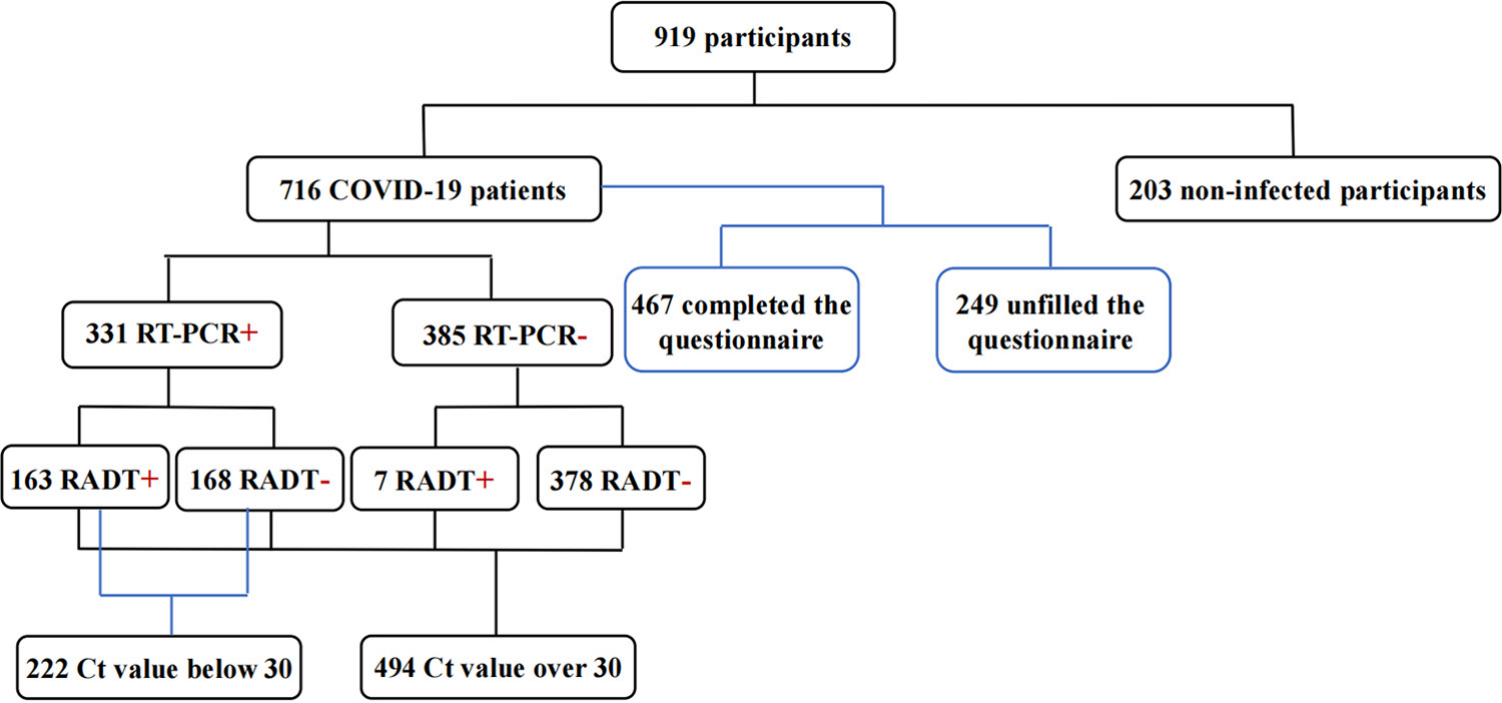
Workflow chart of the data processing. +, positive; −, negative.

A workflow chart of the data processing is shown in Fig. 3. Discrete variables were reported as frequencies and proportions and continuous variables were reported as medians with interquartile ranges to describe the participants’ baseline characteristics. The sensitivity and specificity of the RADT, with 95% confidence intervals (CI), were calculated using RT-quantitative PCR (qPCR) threshold cycle (*C_T_*) values as reference tests. The relationship between the *C_T_* values, sensitivity, and RADT results was assessed using GraphPad Prism (version 8; San Diego, CA, USA). Differences between the two groups were tested using the Mann-Whitney U test. *P* values less than 0.05 were considered statistically significant. The relationship between the *C_T_* values and days after diagnosis was assessed using a segmented linear regression model based on locally estimated scatterplot smoothing (LOESS) regression using R version 3.4.4.
